# Supplementary material for: Drivers of antimicrobial resistance in pig production systems of Uganda
Source: Commun Earth Environ. 2025 Jul 2;6(1):517. doi: 10.1038/s43247-025-02506-8 (PMC12221980; doi:10.1038/s43247-025-02506-8)
Supplement: Supplementary file 2 — Supplementary Information [file 43247_2025_2506_MOESM2_ESM.docx]

**Supplementary material: Drivers of antimicrobial resistance in pig production systems of Uganda**

Adrian Muwonge^1^, Tadeo Kakooza^2^, Paul C. D. Johnson^2^, Lawrence Kisuule^3,4^, Michael Kimaanga^3,4^, Clovice Kankya^3^, Barend Mark de Clare Bronsvoort^1^, Tiziana Lembo^2^

^1^ The Digital One Health Laboratory at The Roslin Institute, R(D)SVS, University of Edinburgh, Easter Bush Campus, Midlothian, EH25 9RG, Scotland

^2^ School of Biodiversity, One Health & Veterinary Medicine, College of Medical, Veterinary & Life Sciences, University of Glasgow, Glasgow G12 8QQ, UK

^2^ Department of Biosecurity Ecosystems and Veterinary Public Health, Makerere University, Kampala, Uganda P.O Box 7062

^4^ Ministry of Agriculture and Animal Industry and Fisheries Uganda, P.O Box 102, Entebbe Plot 16-18, Lugard Avenue, Entebbe

AM: 0000-0002-8579-0384

PJ: 0000-0001-6663-7520

MB: 0000-0002-3271-8485

TL: 0000-0002-6405-1849

**Supplementary Table S1. Summary of samples and metadata collected from 70 households and 20 swine-exposure references during six visits in a one-year study of semi-intensive (peri-urban) and subsistence free-range (rural) pig production systems in Uganda.** Metadata are presented according to the host types, i.e. farmers, swine(SE) and non-swine(NSE) exposure references and pigs. For farmers, we examined predictors such as gender, wealth attainment (marital status and education level), lifestyle-associated factors (smoking habits and alcohol consumption) and medication use. Note that data collected about the pigs comes from questionnaires to the farmer. These included pig breed, management-associated factors (housing, cleaning frequency and feed) and medication use. *On some visits, the farmer or exposure reference did not declare some information which, therefore, unable to record.

| **Variable** | **Categories** | **Household*** | **Pig production system***  “*Kampala and Mubende district”* | | **Sentinel bacteria** |
| --- | --- | --- | --- | --- | --- |
|  |  |  | **Semi-intensive** | **Free-range*** |  |
| **Farmer** | *Levels* | *Participants*  *(N=70)* | *Faecal samples collected*  *(N=386)* | | *Sentinel bacteria*  *(N=296)* |
| Gender* | Female | 22(31.4%) | 56(46.0%) | 65(44.0%) | 100(33.7) |
|  | Male | 48(68.6%) | 140(52.0%) | 125(48.0%) | 196(66.2%) |
| Marital status | Divorced | 3(4.2%) | 3(16.0%) | 15(84.0%) | 10(3.3%) |
|  | Married | 42(60%) | 144(53.0%) | 123(47.0%) | 202(68.2) |
|  | Single | 14(20%) | 40(50.0%) | 40(50.0%) | 66(22.2) |
|  | Widow | 4(5.7%) | 9(42.0%) | 12(58.0%) | 15(5%) |
| Education | None | 2(2.8%) | 2(100.0%) | 0(0.0%) | - |
|  | Primary | 27(38.5%) | 74(50.3%) | 73(49.7%) | 112(37.8%) |
|  | Secondary | 31(44.3%) | 94(54.3%) | 79(45.7%) | 143(48.3) |
|  | Tertiary | 10(14.2%) | 26(40.6%) | 38(59.4%) | 38(12.8%) |
| Smoking status | Yes | 3(4.3%) | 0(0.0%) | 17(100.0%) | 15(5%) |
|  | No | 67(95.7%) | 196(53.0 %) | 173(47.0%) | 281(95%) |
| Alcohol consumption status | Yes | 20(28.5%) | 59(53.1%) | 52(46.9%) | 219(74%) |
|  | No | 50(71.4%) | 137(49.8%) | 138(50.2%) | 77(26%) |
| Antibiotic use | Yes | 15(21.4%) | 44(52.3%) | 40(47.7%) | 232(78.3%) |
|  | No | 55(78.5%) | 152(50.3%) | 150(49.7%) | 64(21.7%) |
| **Swine exposure references*** |  | ***(N=20)*** | ***(N=109)*** |  | ***(N=76)*** |
| Gender* | Female | 4(20%) | 6(30%) | 14(70.0%) | 16(21%) |
|  | Male | 16(80%) | 19(45.2%) | 23(54.8%) | 60(79%) |
| Marital status | Divorced | 3(15%) | 0(0.0%) | 3(100.0%) | 2(3%) |
|  | Married | 12(60%) | 20(38.4%) | 32(61.6%) | 50(75.8) |
|  | Single | 3(15%) | 5(100.0%) | 0(0.0%) | 11(16.6%) |
|  | Window | 2(10%) | 0(0.0%) | 2(100.0%) | 3(4.6%) |
| Exposure to Swine | Low | 10(50%) | 25(47.2%) | 28(52.8%) | 33(43.4%) |
|  | High | 10(50%) | 29(51.7%) | 27(48.3%) | 46(56.6) |
| Education | None | 2(10%) | 2(100.0%) | 0(0.0%) | 3(4%) |
|  | Primary | 9(45%) | 7(31.8%) | 15(69.2%) | 23(30%) |
|  | Secondary | 6(30%) | 10(41.6%) | 14(58.4%) | 25(33%) |
|  | Tertiary | 3(15%) | 6(42.8%) | 8(57.2%) | 15(20%) |
| Smoking status | Yes | 2(10) | 0(0.0%) | 5(100.0%) | 5(7%) |
|  | No | 18(90) | 25(43.8%) | 32(56.2%) | 61(80%) |
| Alcohol consumption status | Yes | 8(60%) | 18(66.6%) | 9(33.4%) | 35(46%) |
|  | No | 12(60%) | 7(20.0%) | 28(80.0%) | 31(41%) |
| Antibiotic Use | No | 57(81%) | 31(86%) | 26(86%) | 57(75%) |
|  | Yes | 9(13%) | 5(14%) | 4(13%) | 9(12%) |
| **Pigs** |  | ***(N=70)*** | ***(N=382)*** | | ***(N=296)*** |
| Antibiotic use* | Yes | 28(40%) | 73(62.3%) | 44(37.7%) | 87(29%) |
|  | No | 42(60%) | 113(48.9%) | 118(51.1%) | 180(61%) |
| Breed | Exotic | 21(30%) | 30(45.4%) | 36(54.6) | 55(19%) |
|  | Local | 14(20%) | 42(56%) | 33(44.0%) | 31(10.4%) |
|  | Mixed | 35(50%) | 114(55.1%) | 93(44.9%) | 171(58%) |
| Housing | Indoor | 45(65%) | 132(52.3%) | 120(47.7%) | 225(76.0%) |
|  | Outdoor | 25 (25%) | 54(56.2%) | 42(43.8%) | 42(14.1%) |
| Feeding | Commercial feed | 5(7.5%) | 11(34.3%) | 21(65.7%) | 23(8%) |
|  | Mixed | 52(74%) | 163(57.2%) | 122(43.8%) | 231(78%) |
|  | Swill | 13(18.5) | 12(38.7%) | 19(61.3%) | 13(4.3) |
| Cleaning frequency | Daily | 32(45.7%) | 97(54.8%) | 80(45.2) | 163(55%) |
|  | Twice a week | 9(12.8) | 30(49.1%) | 31(50.9) | 44(15%) |
|  | Weekly | 19(27.1) | 28(56%) | 22(44.0%) | 35(12%) |
|  | Never | 10(14.4) | 31(51.6%) | 29(58.4%) | 25(8.4%) |

**The relationship between multi-antibiotic resistance and AMR gene copy count**

**Supplementary Figure S1.** The relationship between multi-antibiotic resistance and normalized gene copy number in farmers and their pigs reveals that carriage of *tetQ* is seven-fold higher than that of *tetB*, with significant differences in *tetB* levels between pigs and their farmers. Overall, the association between *ermB* carriage and multidrug resistance is stronger in pigs than in farmers.

**A comparison of phenotypic resistance between sentinel bacteria**

Although *E. coli* and *Klebsiella* belong to the same taxonomic family and exhibit similar resistance profiles, there were subtle differences. For example *Klebsiella* had lower resistance levels per antibiotic compared to *E. coli* (Fig. S2A). Additionally, differences between farmers and their pigs were more pronounced in *Klebsiella* (Fig. S2A). If the rarity of extreme multidrug resistance suggests a fitness cost to sentinel bacteria, this cost appears to be higher for *Klebsiella* (Fig. S2B).

**Supplementary Figure S2** Comparison of phenotypic resistance levels of sentinel bacteria. A. The level is measured as the proportion of resistance (Y-axis) per antibiotic (X-axis). B. The proportion of bacteria exhibiting resistance to the number of antibiotics (X-axis) for “pairs” of farmers and their pigs. The colours refer to the host (blue = pigs; green = farmers). TMPS = trimethoprim/sulfamethoxazole.

**Comparison of phenotypic resistance of “the pairs” and swine exposure references**

There were differences in the prevalence of phenotypic resistance between swine and non-swine exposure references. Resistance was higher in semi-intensive production systems. The proportion of resistant sentinel bacteria was generally lower for exposure references in free-range production systems. This difference was wider between exposure references when examining resistance to streptomycin and tetracycline. We considered a ***swine exposure(SE) reference*** an individual who is in contact with pig gut content much more frequently than the average person, for example a person who is responsible for intestinal evisceration in an abattoir. In contrast to this type of exposure reference, a ***non-swine exposure(NSE) reference*** would be a person who has limited to no contact with pigs due religious beliefs. In general, the proportion of resistant bacteria in SE and NSE references was lower than that in pigs and farmers.

**Supplementary Figure S3.** Comparison of phenotypic resistance levels of swine exposure (SE) references (individuals likely to come into contact with pig intestinal content) and non-swine exposure (NSE) references (individuals at very low risk of coming into contact with pigs) recruited to provide a comparison with study participants (farmer-pig pairs) in Uganda. A & B. Proportion of resistant bacteria (Y-axis) to antibiotics tested (X-axis). C & D. Proportion of bacteria exhibiting resistance to one or more antibiotics (X-axis) for both pairs and references. The reference group shows a similar resistance pattern to the pig-farmer pairs. However, NSE references exhibit higher resistance in semi-intensive/peri-urban settings. SE references and pigs had similar resistance patterns at both mono- (resistance to one antibiotic) and multi- (resistance to multiple antibiotics) resistance levels. The distribution of MDR suggests a fitness cost associated with the multi-resistance phenotype, making it increasingly rare as resistance accumulates. The colours refer to the production system (blue = free-range; red = semi-intensive). Note that b also reflects the dynamics of exposure references. TMPS = trimethoprim/sulfamethoxazole.

**Supplementary Table S2**. Mixed-effect logistic regression model[Model 4] to identify factors associated with phenotypic resistance of *Escherichia coli* and *Klebsiella* species isolated from farmer-pig pairs in semi-intensive and free-range production systems of Uganda. TMPS = trimethoprim/sulfamethoxazole.

|  | **Phenotypic Resistance** | | |
| --- | --- | --- | --- |
| *Predictors* | *Odds Ratios* | *CI* | *p* |
| Production [Free range] | 1 | - | - |
| Production [Free range] | 0.95 | 0.68 – 1.32 | 0.754 |
| Sentinel [*E.coli*] | 1 | - | - |
| Sentinel [*Klebsiella* spp] | 0.52 | 0.42 – 0.64 | **<0.001** |
| Pig breeds [Exotic] | 1 | - | **-** |
| Pig breeds [Mixed] | 0.82 | 0.61 – 1.08 | 0.161 |
| Pig breeds [Local] | 0.39 | 0.25 – 0.60 | **<0.001** |
| Antibtiotic [Ciprofloxacin] | 1 | - | **1** |
| Antibtiotic [Gentamycin] | 1.25 | 0.76 – 2.03 | 0.381 |
| Antibtiotic [Nalidixic acid] | 2.41 | 1.54 – 3.77 | **<0.001** |
| Antibtiotic[Chloramphenicol] | 1.78 | 1.11 – 2.83 | **0.016** |
| Antibtiotic[Streptomycin] | 6.83 | 4.48 – 10.40 | **<0.001** |
| Antibtiotic [TMPS] | 13.89 | 9.14 – 21.11 | **<0.001** |
| Antibtiotic[Tetracycline] | 12.21 | 8.04 – 18.55 | **<0.001** |
| Host [Farmer] | 1 | - | **-** |
| Host [Pig] | 0.91 | 0.72 – 1.15 | 0.418 |
| Visits | 1.15 | 1.02 – 1.31 | **0.024** |
| *tetQ* | 1.24 | 1.10 – 1.40 | **0.001** |
| Production [Free range] ×Host [Pig] | 0.44 | 0.28 – 0.70 | **<0.001** |
| **Random Effects** | | | |
| σ^2^ | 3.29 | | |
| τ_00_ _Sample_ID_ | 0.07 | | |
| τ_00_ _VIZIT_ | 0.04 | | |
| ICC | 0.03 | | |
| N _Sample_ID_ | 65 | | |
| N _VIZIT_ | 6 | | |
| Observations | 2947 | | |
| Marginal R^2^ / Conditional R^2^ | 0.307 / 0.331 | | |

**Estimating an increase in phenotypic resistance**

We used the output from the final model (Table 1, Model 4) to estimate the slope, representing the rate of antibiotic resistance increase to the seven antibiotics over the one-year study period. Although the X-axis in Fig S4 displays six visits, each visit occurred at two-month intervals, effectively scaling the X-axis to 12 months.

**Supplementary Figure S4.** To estimate the rate of increase in antibiotic resistance across the seven investigated antibiotics, we calculated the slope of the fitted line, which represents the rate of increase over the study period. Our analysis suggests that the rate of increase in semi-intensive systems was 1.5 times higher than in free-range settings.

**Factors associated with ABR gene and phenotypic resistance with in exposure references included in the statistical models**

Phenotypic resistance estimates ranged from 4% to 60% for ciprofloxacin and sulfamethoxazole/ trimethoprim, respectively. Resistance was highest and lowest in bacteria isolated from farmers and SE and NSE references, respectively. Farmers had higher gene carriage than the exposure references but generally pigs had fewer genes than farmers and exposure references.

**Supplementary Table S3.** Mixed-effect logistic regression model (Table 1, Model 4) examining the relationship between phenotypic resistance to seven antibiotics of sentinel bacteria *Escherichia coli* and *Klebsiella* isolated from farmers, pigs and swine exposure (individuals likely to come into contact with pig intestinal content) and non-swine (individuals at very low risk of coming into contact with pigs) exposure references (SE Ref and NSE Ref, respectively) in semi-intensive and free-range production systems of Uganda. TMPS = trimethoprim/sulfamethoxazole.

|  | **Phenotypic resistance estimates** | | | |
| --- | --- | --- | --- | --- |
| *Predictors* | *Odds Ratios* | *CI* | *p value* |  |
| Production [Semi-intensive] | 1.42 | 1.00 – 2.01 | **0.050** |  |
| Bacteria [*Klebsiella*] | 0.5 | 0.41 – 0.59 | **<0.001** |  |
| Host [NSE Ref] | 0.54 | 0.29 – 1.03 | 0.060 |  |
| Host [Pig] | 0.50 | 0.35 – 0.70 | **<0.001** |  |
| Host [SE Ref] | 0.41 | 0.21 – 0.81 | **0.010** |  |
| Antibiotic [gentamicin] | 1.47 | 0.98 – 2.22 | 0.063 |  |
| Antibiotic [nalidixic acid] | 2.35 | 1.60 – 3.46 | **<0.001** |  |
| Antibiotic [Chloramphenicol] | 1.42 | 1.01 – 2.09 | **<0.001** |  |
| Antibiotic [streptomycin] | 6.04 | 4.20 – 8.69 | **<0.001** |  |
| Antibiotic [TMPS] | 12.21 | 8.51 – 17.50 | **<0.001** |  |
| Antibiotic [tetracycline] | 11.53 | 8.04 – 16.54 | **<0.001** |  |
| Semi-intensive: NSE Ref | 2.20 | 0.97 – 4.98 | 0.058 |  |
| Semi-intensive: Pig | 1.67 | 1.11 – 2.51 | **0.015** |  |
| Semi-intensive: SE Ref | 1.78 | 0.77 – 4.01 | 0.164 |  |
| **Random Effects** | | | |  |
| σ^2^ | 3.29 | | |  |
| τ_00_ _household_id_ | 0.20 | | |  |
| τ_00_ _TIMEZ_ | 0.10 | | |  |
| ICC | 0.09 | | |  |
| N _household_id_ | 136 | | |  |
| N _TIMEZ_ | 6 | | |  |
| Observations | 3388 | | |  |
| Marginal R^2^ / Conditional R^2^ | 0.27/ 0.33 | | |  |

**Supplementary Table S4.** Mixed-effect logistic regression model (Table 1, Model 1B) examining factors associated with AMR phenotype of *Escherichia coli* and *Klebsiella* . Here, the AMR phenotype is for individual antibiotics with R^2^ greater than 12%.

|  | **Trimethoprim Sulfamethoxazole** | | | **Streptomycin** | | | **Tetracycline** | | | **Chloramphenicol** | | |
| --- | --- | --- | --- | --- | --- | --- | --- | --- | --- | --- | --- | --- |
| *Predictors* | *Odds Ratios* | *CI* | *p* | *Odds Ratios* | *CI* | *P* | *Odds Ratios* | *CI* | *p* | *Odds Ratios* | *CI* | *p* |
| Bacteria [Klebsiella] | 0.35 | 0.23 – 0.53 | **<0.001** | 0.37 | 0.22 – 0.65 | **<0.001** | 0.36 | 0.23 – 0.57 | **<0.001** | 1.10 | 0.61 – 2.02 | 0.746 |
| Pig breed [Mixed] | 0.81 | 0.48 – 1.36 | 0.422 |  |  |  | 0.63 | 0.36 – 1.10 | 0.105 |  |  |  |
| Pig breed [Local] | 0.44 | 0.22 – 0.85 | **0.015** |  |  |  | 0.21 | 0.09 – 0.46 | **<0.001** |  |  |  |
| Host [Pig] | 0.59 | 0.40 – 0.89 | **0.011** | 1.02 | 0.41 – 2.51 | 0.974 | 0.66 | 0.44 – 1.00 | 0.050 | 0.62 | 0.34 – 1.13 | 0.119 |
| Visit | 1.05 | 0.90 – 1.22 | 0.557 | 2.16 | 1.38 – 3.37 | **0.001** | 1.19 | 0.88 – 1.59 | 0.258 |  |  |  |
| *tetQ* | 1.23 | 0.90 – 1.66 | 0.189 | 1.48 | 1.00 – 2.18 | **0.047** | 1.97 | 1.22 – 3.18 | **0.006** |  |  |  |
| Pig treatment in the last 14 days [YES] | 0.95 | 0.62 – 1.47 | 0.816 |  |  |  | 1.11 | 0.70 – 1.76 | 0.666 | 0.47 | 0.23 – 0.99 | **0.048** |
| *tetB* | 1.23 | 1.02 – 1.47 | **0.027** | 0.87 | 0.68 – 1.12 | 0.284 | 0.93 | 0.75 – 1.17 | 0.553 | 1.33 | 1.04 – 1.71 | **0.025** |
| Production[Semi intensive] |  |  |  | 2.75 | 1.16 – 6.51 | **0.022** | 1.23 | 0.76 – 2.00 | 0.400 | 1.95 | 0.75 – 5.06 | 0.169 |
| *ermB* |  |  |  | 0.91 | 0.73 – 1.15 | 0.435 |  |  |  | 0.98 | 0.78 – 1.23 | 0.859 |
| Production[Semi intensive] × Host[Pig] |  |  |  | 1.04 | 0.35 – 3.08 | 0.944 |  |  |  |  |  |  |
| Farmer treatment in the last 14 days [Yes] |  |  |  |  |  |  |  |  |  |  |  |  |
| Pig house cleaning frequency[Never] |  |  |  |  |  |  |  |  |  | 0.80 | 0.19 – 3.38 | 0.762 |
| Pig house cleaning frequency[Twice] |  |  |  |  |  |  |  |  |  | 0.45 | 0.13 – 1.57 | 0.213 |
| Pig house cleaning frequency[Weekly] |  |  |  |  |  |  |  |  |  | 1.59 | 0.55 – 4.57 | 0.391 |
| σ^2^ | 3.29 | | | 3.29 | | | 3.29 | | | 3.29 | | |
| τ_00_ | 0.00 _Subcounty_ | | | 0.15 _Subcounty_ | | | 0.00 _Subcounty_ | | | 0.00 _Subcounty_ | | |
|  | 0.00 _Visit*_ | | | 0.63 _Visit*_ | | | 0.26 _Visit*_ | | | 0.00 _Visit*_ | | |
| ICC | 0.10 | | | 0.19 | | | 0.07 | | | 0.08 | | |
| N | 6 _Visit*_ | | | 6 _Visit*_ | | | 6 _V Visit*_ | | | 6 _Visit*_ | | |
|  | 10 _Subcounty_ | | | 10 _Subcounty_ | | | 10 _Subcounty_ | | | 10 _Subcounty_ | | |
| Observations | 465 | | | 452 | | | 465 | | | 446 | | |
| Marginal R^2^ / Conditional R^2^ | 0.150 /0.16 | | | 0.378 /0.497 | | | 0.203 / 0.261 | | | 0.153/0.17 | | |

**Supplementary Table S5.** Mixed-effect logistic regression model [Table 1, Model 1B] examining factors associated with AMR phenotype of *Escherichia coli*. Here, the AMR phenotype is for individual antibiotics with R^2^ greater than 12%. Due to reduced degrees of freedom none of the *Klebsiella* models met this criterion.

|  | **Tetracycline** | | | **Streptomycin** | | | **Gentamycin** | | |
| --- | --- | --- | --- | --- | --- | --- | --- | --- | --- |
| *Predictors* | *Odds Ratios* | *CI* | *p* | *Odds Ratios* | *CI* | *p* | *Odds Ratios* | *CI* | *P* |
| (Intercept) | 2.56 | 0.52 – 13.53 | 0.248 | 0.61 | 0.27 – 1.37 | 0.231 | 0.09 | 0.02 – 0.36 | **0.002** |
| Production[Semi-intensive] | 2.19 | 1.08 – 4.56 | **0.032** | 3.00 | 1.68 – 5.47 | **<0.001** | 0.90 | 0.34 – 2.54 | 0.838 |
| Host [Pig] | 0.75 | 0.38 – 1.45 | 0.393 | 1.24 | 0.72 – 2.14 | 0.445 | 1.20 | 0.60 – 2.44 | 0.609 |
| Visit[4] | 0.88 | 0.33 – 2.36 | 0.804 | 1.03 | 0.47 – 2.21 | 0.946 | 1.22 | 0.28 – 6.20 | 0.796 |
| Visit[1] | 0.11 | 0.03 – 0.36 | **<0.001** | 0.06 | 0.02 – 0.19 | **<0.001** | 12.06 | 3.20 – 60.38 | **0.001** |
| Visit[6] | 0.38 | 0.10 – 1.36 | 0.150 | 0.84 | 0.36 – 1.95 | 0.680 | 1.20 | 0.21 – 6.92 | 0.831 |
| Visit[3] | 0.50 | 0.18 – 1.37 | 0.180 | 0.10 | 0.04 – 0.25 | **<0.001** | 6.95 | 2.10 – 31.75 | **0.004** |
| Visit[2] | 0.14 | 0.02 – 0.62 | **0.020** | 0.03 | 0.01 – 0.10 | **<0.001** | 1.24 | 0.22 – 7.12 | 0.801 |
| Pig treatment in the last 14 days[Yes] | 1.61 | 0.72 – 3.62 | 0.248 | 0.64 | 0.33 – 1.23 | 0.183 |  |  |  |
| Farmer treatment in the last 14 days [Yes] | 0.82 | 0.33 – 1.95 | 0.666 |  |  |  | 0.31 | 0.09 – 0.90 | **0.045** |
| Farmer marital status [Married] | 0.29 | 0.06 – 1.33 | 0.112 |  |  |  |  |  |  |
| Farmer marital Status [Single] | 0.11 | 0.02 – 0.60 | **0.012** |  |  |  |  |  |  |
| Farmer marital Status [Widow] | 0.22 | 0.03 – 1.51 | 0.131 |  |  |  |  |  |  |
| Farmer education [Secondary] |  |  |  | 1.93 | 1.07 – 3.51 | **0.030** | 0.68 | 0.32 – 1.42 | 0.306 |
| Farmer education [Tertiary] |  |  |  | 2.41 | 0.86 – 6.69 | 0.091 | 0.83 | 0.24 – 2.51 | 0.759 |
| Pig cleaning frequency[Never] |  |  |  |  |  |  | 0.17 | 0.02 – 0.88 | 0.051 |
| Pig cleaning frequency [Twice a week] |  |  |  |  |  |  | 0.67 | 0.21 – 1.95 | 0.485 |
| Pig cleaning frequency[Weekly] |  |  |  |  |  |  | 0.67 | 0.18 – 2.37 | 0.535 |
| Observations | 202 | | | 341 | | | 341 | | |
| R^2^ Tjur | 0.144 | | | 0.360 | | | 0.142 | | |

**Temporal characteristics of AMR**

**Supplementary Figure S5.** Temporal shift in phenotypic resistance across the study period (X axis). Here, we assessed the phenotypic resistance to each antibiotic over time for each place/production system. There was a significant increase in the proportion of resistance over time to chloramphenicol and streptomycin among farmers and pigs in semi-intensive production systems. This relationship is also shown in the regression models in **Supplementary Tables S4 & 5.**

**Supplementary Table S6.** Generalized linear mixed models (Table 1, Model 3) of gene carriage of a farmer at time point (t) against gene carriage of their pig at time point (t+1). The model shows that there is no influence of one host on the carriage of another in any of the studied systems. ICC = intraclass correlation coefficient (ICC) (= 0.21, R^2^= 0.74).

| **Response variable** | **Explanatory variable** | **Estimate** | **CI** | **P Value** | **Variance** |
| --- | --- | --- | --- | --- | --- |
| Log10(copy number of genes from Farmer) at (t) |  |  |  |  |  |
|  | Log10 (copy number of genes from Pig) at (t+1) | -0.02 | -0.12 – 0.08 | 0.721 |  |
|  | *dfra1* | 1(ref) | - | - |  |
|  | *ermB* | -0.16 | -0.81 – 0.49 | 0.623 |  |
|  | *tetB* | -0.33 | -0.99 – 0.32 | 0.318 |  |
|  | *tetQ* | 4.34 | 3.56 – 5.12 | **<0.001** |  |
|  | Free-range | 1(ref) | - | - |  |
|  | Semi-intensive | 0.46 | -0.24 – 1.15 | 0.197 |  |
|  | *ermB* : Semi-intensive | 0.55 | -0.18 – 1.28 | 0.141 |  |
|  | *tetB* : Semi-intensive | -0.07 | -0.81 – 0.67 | 0.855 |  |
|  | *tetQ*: Semi-intensive | -0.43 | -1.15 – 0.29 | 0.238 |  |
| Random effect | Farm |  |  |  | 0.31 |

**Supplementary Table S7**. Mixed-effect linear regression model (Table 1, Model 4) to identify factors associated with gene carriage in farmers and their pigs in semi- intensive and free-range production systems of Uganda. ABR = antibiotic resistance.

| Variables | Levels | Estimate  (95%CI) | P Value | Variance |
| --- | --- | --- | --- | --- |
| Fixed effects |  |  |  |  |
| Production | Semi-intensive | 1 (ref.) |  |  |
|  | Free-range | 0.49 (0.25-0.72) | P<0.001 |  |
| Host | Farmer | 1 (ref.) |  |  |
|  | Pig | -0.64 (-0.84- -0.45) | P<0.001 |  |
| ABR genes | *dfra1* | 1 (ref.) |  |  |
|  | *ermB* | 0.29 (0.14-0.45) | P<0.001 |  |
|  | *tetB* | -0.72 (-0.87-0.56) | P<0.001 |  |
|  | *tetQ* | 3.97 (3.81-4.12) | P<0.001 |  |
|  |  |  |  |  |
| Interaction term | Semi-intensive*pig | 0.27(0.02-0.51) | 0.031 |  |
| Random effects |  |  |  |  |
| Farm |  |  |  | 0.10 |
| Visit |  |  |  | 0.16 |

**Determining the structure and importance of drivers of AMR using the conditional inference trees**

This approach is a non-parametric decision tree based on conditional inference principles. Here, the tree bifurcation is informed by p-values from a permutational distribution. The root represents the most significant predictor. Antibiotic type is the strongest predictor of resistance, with antibiotics splitting into high- and low-resistance groups. The low-resistance group (ciprofloxacin, nalidixic acid, chloramphenicol) offers limited explanatory power for other predictors. Most of the variable partitioning occurs in the high-resistance group, where the type of sentinel bacteria—particularly *E. coli*—emerges as the second most important factor.

For instance, production system plays a key role: In free-range pigs, no antibiotic use corresponds to low resistance. In semi-intensive systems, no antibiotic use in local breeds is linked to absence of resistance, while exotic breeds and mixed medication (including human antibiotic use) show lower resistance. Unlike the conventional regression models (1-4), this acyclic approach highlights AMU, particularly in pigs, as a strong explanatory variable for resistance within different production systems.

**Supplementary Figure S6.** Condition tree inferencing was also used to determine the true effect of predictors used in the Models 1-4 (Table 1).

**Supplementary Table S8.** Mixed-effect linear regression model examining the relationship between gene carriage among host (i.e. farmer/pig), and swine exposure (individuals likely to come into contact with pig intestinal content) and non-swine exposure (individuals at very low risk of coming into contact with pigs) exposure references (SE Ref and NSE Ref, respectively), in semi-intensive and free-range production systems of Uganda.

|  | **log10(AMR gene copy number)** | | |
| --- | --- | --- | --- |
| *Predictors* | *Estimates* | *95% CI* | *p value* |
| Host [Pig] | 0.02 | -0.09 – 0.12 | 0.768 |
| Host [SE Ref] | 1.89 | 1.24 – 2.53 | **<0.001** |
| Host [NSE Ref] | 1.76 | 1.12 – 2.41 | **<0.001** |
| Time[Visits] | 0.04 | -0.18 – 0.26 | 0.738 |
| Genes [*ermB*] | -0.47 | -0.58 – -0.36 | **<0.001** |
| Genes [*tetB*] | -0.56 | -0.58 – -0.35 | **<0.001** |
| Genes [*dfra*] | -1.55 | -1.78 – -1.31 | **<0.001** |
| Production [Semi-intensive] | 0.78 | 0.44 – 1.12 | **<0.001** |
| Pig: Semi-intensive | -0.02 | -0.15 – 0.11 | 0.737 |
| SE Ref: Semi-intensive | -0.90 | -1.74 – -0.07 | **0.034** |
| *ermB*: Semi-intensive | 0.12 | -0.02 – 0.26 | **0.087** |
| *tetB*: Semi-intensive | 0.35 | 0.21 – 0.48 | **<0.001** |
| *dfra1*: Semi-intensive | 0.24 | -0.05 – 0.54 | 0.108 |
| **Random Effects** | | | |
| σ^2^ | 1.97 | | |
| τ_00_ _hh_id_ | 0.43 | | |
| τ_00_ _TIME_ | 0.22 | | |
| ICC | 0.25 | | |
| N _hh_id_ | 85 | | |
| N _TIME_ | 6 | | |
| Observations | 11039 | | |
| Marginal R^2^ / Conditional R^2^ | 0.122 / 0.338 | | |

Swine exposure ref

Non-Swine exposure ref

**Supplementary Figure S7. G**ene carriage of low and high exposure references as detected from samples collected in the study sites. We targeted antibacterial resistance genes encoding for resistance to commonly used antibacterials: tetracyclines (*tetB* and *tetQ*), tylosin (*ermB*) and trimethoprim (*dfrA1*).

**Determining importance of factors associated with AMR-gene carriage by farmers and their pigs**

**A**

**B**

**C**

**D**

**Supplementary Figure S8.** Condition tree inferencing was also used to determine the true effect of predictors for gene carriage by farmer and pigs. The strongest predictor of *tetQ* (panel A), *ermB* (Panel C) and *dfra1*(Panel D) is frequency of cleaning, whilst for *tetB* (Panel B) is production system.

**Inference of transmission at the human animal interface**

**Multi-resistance phenotype**

Our assumption was that the sharing of multidrug resistance (MDR) traits is a rare event at the household level and even more rare between pigs in different sub-counties and districts. Therefore, when encountered it likely represents a transmission event. We used sixteen unique MDR patterns to investigate this question. The most common MDR pattern was resistance to streptomycin, tetracycline and trimethoprim/sulfamethoxazole which are the most used antibiotics in these livestock production systems.

**Supplementary Figure S9**. Prevalence of sixteen unique multidrug resistance (MDR) profiles identified in this study colored by production system and host. MDR levels were much higher in semi-intensive(peri-urban) production systems and slightly higher among farmers. The dotted line represents the cut-off for downstream analysis for the liberal (<10%) and conservative (>10%) inferences of transmission. Note that peri-urban= semi-intensive and rural= free range. TMPS = trimethoprim/sulfamethoxazole.

**Supplementary Table S9.** The proportion of shared MDR strains between a farmer and their pig at farm level, and within and between production systems.

| *Comparison* | | *Transmission estimates* | |
| --- | --- | --- | --- |
|  |  | Conservative  N=490 | Liberal  N=1850 |
| Farmers and pigs between production systems | (26)11% | | (66)11.1 % |
| Farmers and pigs within production systems | | (12)5.7 % | (330)30.7% |
| Farmers and pigs  on farms | | (10)23.8% | (68)38.2 % |
| ***Prevalence of transmission events*** | | (48) 9.8% | (464) 25.1% |

*χ^2^=13.836 · df=2 · Cramer's V=0.168 · Fisher's p=0.001 (Conservative estimates)*

*χ^2^=96.422 · df=2 · Cramer's V=0.228 · p=0.000 (Liberal estimates). Note here that we have used the liberal estimate for the figure 6A*

**Supplementary Figure S10.** Frequency of transmission events at different spatial scales, farm/household, within and between production systems. This figure relates to Supplementary Table S7 above and shows the prevalence using conservative estimates.

**Materials and Methods**

**Study site**

This study was conducted in two districts of Uganda, Kampala and Mubende, - five sub-counties each - representing peri-urban (semi-intensive) and rural (/free-range) livestock production systems, respectively.

**Supplementary Figure S11.** Map of the study area showing the five sub-counties in each of the peri-urban (semi-intensive livestock production systems) and rural (free-range livestock production systems) districts (Kampala and Mubende, respectively) of Uganda where samples and data analysed in this study were

collected from pairs of pigs and farmers. The pink bubbles represent all the visits to a household during the study jittered at 0.001 x 0.001 for data governance reasons**.**


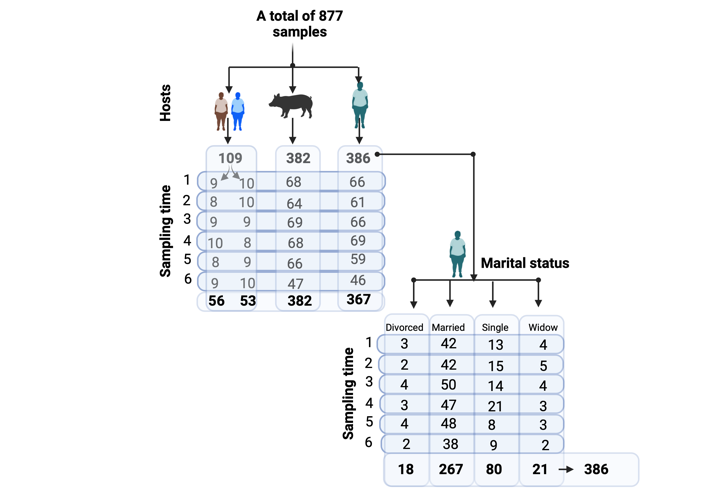


Swine exposure ref

**Supplementary Figure S12.** **Participant recruitment and sample collection across time.** The flow chart shows the number of study participants recruited in this study, as well as the samples collected from each group at each of the six sampling points and marital status information for the farmers interviewed. We illustrate host and marital status information available for each of the farms recruited in order to elucidate any disparities in numbers that arose due to missing data. For instance, the brown, blue and green human represent high, low exposure references and farmers. While marital status information was available for all 386 farmers from whom samples were collected, the host label was missing for 19 samples, resulting in a discrepancy. The figure also displays the samples collected across these variables for the six sampling points, facilitating an evaluation of the temporal aspects in this longitudinal cohort study. This aims to highlight the discrepancies that can arise due to missingness in data.

**Phenotypic antibiotic resistance diffusion disc interpretation**

Disc diameters were interpreted using the European Committee on Antimicrobial Susceptibility Testing system ([EUCAST](https://www.eucast.org/clinical_breakpoints)) to assign a status of “S”, “I” and “R” to indicate Susceptible, Intermediate and Resistant sentinel bacteria, respectively. We then extracted an aliquot of high molecular grade DNA for downstream analyses.

**DNA extraction and quantification**

DNA extraction was performed using the Ultraclean Soil DNA Isolation Kit (MO BIO Laboratories). DNA quantification and quality checks were conducted on each sample extract using the Qubit fluorometer. Antibiotic resistance gene copy numbers were normalised using the 16S rRNA gene copy number which was used as a proxy for the number of bacterial cells per sample. This experiment was done using our recently published in-house methodology^1^. The gene count was normalised by dividing the gene copy number of each gene by the total bacterial load in each sample. So, in effect, this outcome variable approximates the gene copy number per bacterium.

**References**

1. Pollock J, Muwonge A, Hutchings MR, et al. Resistance to change: AMR gene dynamics on a commercial pig farm with high antimicrobial usage. *Sci Rep* 2020; **10**: 1708.
