# Supplementary material for: Drivers of antimicrobial resistance in pig production systems of Uganda
Source: Commun Earth Environ. 2025 Jul 2;6(1):517. doi: 10.1038/s43247-025-02506-8 (PMC12221980; doi:10.1038/s43247-025-02506-8)
Supplement: Supplementary file 3 — Reporting Summary [file 43247_2025_2506_MOESM3_ESM.pdf]

nature portfolio

Corresponding author(s): ADRIAN MUWONGE

Last updated by author(s): May 30, 2025

Reporting Summary

Nature Portfolio wishes to improve the reproducibility of the work that we publish. This form provides structure for consistency and transparency in reporting. For further information on Nature Portfolio policies, see our Editorial Policies and the Editorial Policy Checklist.

Statistics

For all statistical analyses, confirm that the following items are present in the figure legend, table legend, main text, or Methods section.

n/a	Confirmed	
☐	☒	The exact sample size (n) for each experimental group/condition, given as a discrete number and unit of measurement
☐	☒	A statement on whether measurements were taken from distinct samples or whether the same sample was measured repeatedly
☐	☒	The statistical test(s) used AND whether they are one- or two-sided<br>*Only common tests should be described solely by name; describe more complex techniques in the Methods section.*
☐	☒	A description of all covariates tested
☐	☒	A description of any assumptions or corrections, such as tests of normality and adjustment for multiple comparisons
☐	☒	A full description of the statistical parameters including central tendency (e.g. means) or other basic estimates (e.g. regression coefficient) AND variation (e.g. standard deviation) or associated estimates of uncertainty (e.g. confidence intervals)
☐	☒	For null hypothesis testing, the test statistic (e.g. F, t, r) with confidence intervals, effect sizes, degrees of freedom and P value noted<br>*Give P values as exact values whenever suitable.*
☒	☐	For Bayesian analysis, information on the choice of priors and Markov chain Monte Carlo settings
☐	☒	For hierarchical and complex designs, identification of the appropriate level for tests and full reporting of outcomes
☐	☒	Estimates of effect sizes (e.g. Cohen's d, Pearson's r), indicating how they were calculated

Our web collection on statistics for biologists contains articles on many of the points above.

Software and code

Policy information about availability of computer code

Data collection	KOBO Collect
Data analysis	R statistics

For manuscripts utilizing custom algorithms or software that are central to the research but not yet described in published literature, software must be made available to editors and reviewers. We strongly encourage code deposition in a community repository (e.g. GitHub). See the Nature Portfolio guidelines for submitting code & software for further information.

Data

Policy information about availability of data

All manuscripts must include a data availability statement. This statement should provide the following information, where applicable:

- Accession codes, unique identifiers, or web links for publicly available datasets
- A description of any restrictions on data availability
- For clinical datasets or third party data, please ensure that the statement adheres to our policy

The datasets and R-code used for analysis are accessible at https://datashare.ed.ac.uk/handle/10283/8847 or https://doi.org/10.7488/ds/7792

Research involving human participants, their data, or biological material

Policy information about studies with human participants or human data. See also policy information about sex, gender (identity/presentation), and sexual orientation and race, ethnicity and racism.

Reporting on sex and gender	The Gender of farmers was captured and reported in Table S1, we use appropriate terms to report on them where they were relevant in this study
Reporting on race, ethnicity, or other socially relevant groupings	The study is conducted in Uganda in rural and urban on relevant covariates collected and used in the analysis. We did not include ethnicity as part of the variables as our hypothesis did not deem this relevant to the dynamics under investigation
Population characteristics	Farmers were included as part of farm selection process as described below. Sows were used as they remain on farms for reproduction and long-term management. The pig production categorization was based on factors such as stocking density, access to land resources, disposable income, access to improved breeding inputs, supplementation, market access and productivity
Recruitment	Farms were selected from a list provided by the district production office, these were then visited to seek consent to participate. If the farmers did not want to participate then they were not included.
Ethics oversight	Ethical approval was obtained from the: (1) School of Biomedical Sciences, Makerere University College of Health Sciences (REF-SBS-140); (2) Uganda National Council of Science and Technology (UNCST REF: HS103ES); and (3) Easter Bush Veterinary and Human Ethical Review Committees, University of Edinburgh (VERC-82-17 & HERC-133-17). Informed consent was obtained in writing from each participant. Therefore, this study was conducted in compliance with all relevant ethical regulations.

Note that full information on the approval of the study protocol must also be provided in the manuscript.

Field-specific reporting

Please select the one below that is the best fit for your research. If you are not sure, read the appropriate sections before making your selection.

☒ Life sciences ☐ Behavioural & social sciences ☐ Ecological, evolutionary & environmental sciences

For a reference copy of the document with all sections, see nature.com/documents/nr-reporting-summary-flat.pdf

Life sciences study design

All studies must disclose on these points even when the disclosure is negative.

Sample size	Sample-size estimation: Thirty-five farmer-pig pairs were followed per production system. To estimate this sample size, we assumed an AMR prevalence risk ratio of 5.5 with 80% power at the 5% significance level, based on cross-sectional studies with 38% and 10% average prevalence in high- (semi-intensive) and low- (free-range) AMU systems, respectively. We accounted for a 10% attrition rate and clustering (design effect) and included both high-exposure (an abattoir worker handling pig gut evisceration) and low-exposure (individuals with no pig contact due to religious or cultural taboos) swine reference groups in each of the five sub-counties where the pairs resided . A total of 840 and 120 samples were expected from 70 pairs and 20 swine exposure references (10 swine and 10 non-swine exposure references) across six study visits
Data exclusions	We do not have any data exclusion scenarios
Replication	This was a longitudinal study where we followed farmers for one year. We has a farmer and pig per household, which were visited every two months. So for a total of 6 visits. We have in general six replicate per farm but each represents a different time point
Randomization	The selection of farmer is based on a list of farmers generated by the district production department. Farms were randomly selected from this list. If the farmers did not want to participate, we randomly generated another ID for the farm
Blinding	n/a

Reporting for specific materials, systems and methods

We require information from authors about some types of materials, experimental systems and methods used in many studies. Here, indicate whether each material, system or method listed is relevant to your study. If you are not sure if a list item applies to your research, read the appropriate section before selecting a response.

Materials & experimental systems

n/a	Involved in the study
☒	☐ Antibodies
☒	☐ Eukaryotic cell lines
☒	☐ Palaeontology and archaeology
☐	☒ Animals and other organisms
☒	☐ Clinical data
☒	☐ Dual use research of concern
☒	☐ Plants

Methods

n/a	Involved in the study
☐	☐ ChIP-seq
☐	☐ Flow cytometry
☐	☐ MRI-based neuroimaging

Animals and other research organisms

Policy information about studies involving animals; ARRIVE guidelines recommended for reporting animal research, and Sex and Gender in Research

Laboratory animals

For laboratory animals, report species, strain and age OR state that the study did not involve laboratory animals.

Wild animals

Provide details on animals observed in or captured in the field; report species and age where possible. Describe how animals were caught and transported and what happened to captive animals after the study (if killed, explain why and describe method; if released, say where and when) OR state that the study did not involve wild animals.

Reporting on sex

The sex of animal here is not relevant as all pigs included were sows

Field-collected samples

Faecal samples, collected using a stool collection kit from Farmer and their pigs with consent. The farmer fecal sample was collected by the Farmer the day before our visit. The sample from the sow was collected by a veterinarian part of the research team. These were then submitted to the Molecular Biology Laboratory at the Schools of Biomedical Sciences of Makerere University (Kampala, Uganda) for culture, sensitivity testing and DNA extraction. An aliquot was sent to the Makerere Central Veterinary laboratory for bacterial culture. DNA was sent to the Digital One Health Laboratory, Roslin Institute, University of Edinburgh for down-stream qPCR analysis.

Ethics oversight

Ethical approval was obtained from the: (1) School of Biomedical Sciences, Makerere University College of Health Sciences (REF-SBS-140); (2) Uganda National Council of Science and Technology (UNCST REF: HS103ES); and (3) Easter Bush Veterinary and Human Ethical Review Committees, University of Edinburgh (VERC-82-17 & HERC-133-17). Informed consent was obtained in writing from each participant.

Note that full information on the approval of the study protocol must also be provided in the manuscript.

Plants

Seed stocks

Report on the source of all seed stocks or other plant material used. If applicable, state the seed stock centre and catalogue number. If plant specimens were collected from the field, describe the collection location, date and sampling procedures.

Novel plant genotypes

Describe the methods by which all novel plant genotypes were produced. This includes those generated by transgenic approaches, gene editing, chemical/radiation-based mutagenesis and hybridization. For transgenic lines, describe the transformation method, the number of independent lines analyzed and the generation upon which experiments were performed. For gene-edited lines, describe the editor used, the endogenous sequence targeted for editing, the targeting guide RNA sequence (if applicable) and how the editor was applied.

Authentication

Describe any authentication procedures for each seed stock used or novel genotype generated. Describe any experiments used to assess the effect of a mutation and, where applicable, how potential secondary effects (e.g. second site T-DNA insertions, mosiacism, off-target gene editing) were examined.

ChIP-seq

Data deposition

☐ Confirm that both raw and final processed data have been deposited in a public database such as GEO.

☐ Confirm that you have deposited or provided access to graph files (e.g. BED files) for the called peaks.

Data access links
May remain private before publication.

For "Initial submission" or "Revised version" documents, provide reviewer access links. For your "Final submission" document, provide a link to the deposited data.

Files in database submission

Provide a list of all files available in the database submission.

Genome browser session
(e.g. UCSC)

Provide a link to an anonymized genome browser session for "Initial submission" and "Revised version" documents only, to enable peer review. Write "no longer applicable" for "Final submission" documents.

Methodology

Replicates	*Describe the experimental replicates, specifying number, type and replicate agreement.*
Sequencing depth	*Describe the sequencing depth for each experiment, providing the total number of reads, uniquely mapped reads, length of reads and whether they were paired- or single-end.*
Antibodies	*Describe the antibodies used for the ChIP-seq experiments; as applicable, provide supplier name, catalog number, clone name, and lot number.*
Peak calling parameters	*Specify the command line program and parameters used for read mapping and peak calling, including the ChIP, control and index files used.*
Data quality	*Describe the methods used to ensure data quality in full detail, including how many peaks are at FDR 5% and above 5-fold enrichment.*
Software	*Describe the software used to collect and analyze the ChIP-seq data. For custom code that has been deposited into a community repository, provide accession details.*

Flow Cytometry

Plots

Confirm that:

☐ The axis labels state the marker and fluorochrome used (e.g. CD4-FITC).

☐ The axis scales are clearly visible. Include numbers along axes only for bottom left plot of group (a 'group' is an analysis of identical markers).

☐ All plots are contour plots with outliers or pseudocolor plots.

☐ A numerical value for number of cells or percentage (with statistics) is provided.

Methodology

Sample preparation	*Describe the sample preparation, detailing the biological source of the cells and any tissue processing steps used.*
Instrument	*Identify the instrument used for data collection, specifying make and model number.*
Software	*Describe the software used to collect and analyze the flow cytometry data. For custom code that has been deposited into a community repository, provide accession details.*
Cell population abundance	*Describe the abundance of the relevant cell populations within post-sort fractions, providing details on the purity of the samples and how it was determined.*
Gating strategy	*Describe the gating strategy used for all relevant experiments, specifying the preliminary FSC/SSC gates of the starting cell population, indicating where boundaries between "positive" and "negative" staining cell populations are defined.*

☐ Tick this box to confirm that a figure exemplifying the gating strategy is provided in the Supplementary Information.

Magnetic resonance imaging

Experimental design

Design type	*Indicate task or resting state; event-related or block design.*
Design specifications	*Specify the number of blocks, trials or experimental units per session and/or subject, and specify the length of each trial or block (if trials are blocked) and interval between trials.*
Behavioral performance measures	*State number and/or type of variables recorded (e.g. correct button press, response time) and what statistics were used to establish that the subjects were performing the task as expected (e.g. mean, range, and/or standard deviation across subjects).*

Acquisition

Imaging type(s)

Specify: functional, structural, diffusion, perfusion.

Field strength

Specify in Tesla

Sequence & imaging parameters

Specify the pulse sequence type (gradient echo, spin echo, etc.), imaging type (EPI, spiral, etc.), field of view, matrix size, slice thickness, orientation and TE/TR/flip angle.

Area of acquisition

State whether a whole brain scan was used OR define the area of acquisition, describing how the region was determined.

Diffusion MRI ☐ Used ☒ Not used

Preprocessing

Preprocessing software

Provide detail on software version and revision number and on specific parameters (model/functions, brain extraction, segmentation, smoothing kernel size, etc.).

Normalization

If data were normalized/standardized, describe the approach(es): specify linear or non-linear and define image types used for transformation OR indicate that data were not normalized and explain rationale for lack of normalization.

Normalization template

Describe the template used for normalization/transformation, specifying subject space or group standardized space (e.g. original Talairach, MNI305, ICBM152) OR indicate that the data were not normalized.

Noise and artifact removal

Describe your procedure(s) for artifact and structured noise removal, specifying motion parameters, tissue signals and physiological signals (heart rate, respiration).

Volume censoring

Define your software and/or method and criteria for volume censoring, and state the extent of such censoring.

Statistical modeling & inference

Model type and settings

Specify type (mass univariate, multivariate, RSA, predictive, etc.) and describe essential details of the model at the first and second levels (e.g. fixed, random or mixed effects; drift or auto-correlation).

Effect(s) tested

Define precise effect in terms of the task or stimulus conditions instead of psychological concepts and indicate whether ANOVA or factorial designs were used.

Specify type of analysis: ☐ Whole brain ☐ ROI-based ☐ Both

Statistic type for inference

(See Eklund et al. 2016)

Specify voxel-wise or cluster-wise and report all relevant parameters for cluster-wise methods.

Correction

Describe the type of correction and how it is obtained for multiple comparisons (e.g. FWE, FDR, permutation or Monte Carlo).

Models & analysis

n/a	Involved in the study
☒	☐ Functional and/or effective connectivity
☒	☐ Graph analysis
☐	☒ Multivariate modeling or predictive analysis

Multivariate modeling and predictive analysis

Depending on the model we used different variable, please note that we have 19 models including logistic and linear model a shown in table 1. We used variables such as -Fixed terms Bacterium species (Escherichia coli /Klebsiella), production system (semi-intensive/free-range), host species (farmer/pig), host species (farmer/pig), visit , pig breed, cleaning, AMR gene and treatment. Random Variables such as Farm ID‡, visit‡, antibiotic type (tetracycline/ ciprofloxacin/ gentamycin/Chloramphenicol/ streptomycin/ nalidixic acid/ trimethoprim-sulfamethoxazole)
